# Preparation of microcrystalline cellulose from *Rabdosia rubescens* residue and study on its membrane properties

**DOI:** 10.1038/s41598-021-98645-x

**Published:** 2021-09-23

**Authors:** Meng Li, Tong Wei, Chaoyi Qian, Zhengyong Liang

**Affiliations:** 1 School of Chemical Engineering, Zhengzhou University, Zhengzhou, Henan China; 2 Zhengzhou University-Jiyuan Research Institute, Jiyuan, Henan China

**Keywords:** Biomaterials, Sustainability

## Abstract

Microcrystalline cellulose (MCC) was prepared easily from *Rabdosia rubescens* residue to realize the efficient utilization of waste resources. The yield was about 95.03% under the optimal conditions. Then, MCC membrane was prepared by phase transformation method and its structure and mechanical properties were studied systemically. The results showed the cellulose crystal structure changed from type I to type II in the process of forming membrane, and the thermal stability decreased simultaneously. The content of MCC in casting solution has great influence on the mechanical properties of membranes. The higher the content of MCC, the better the comprehensive mechanical properties of the membranes is. When MCC content is 9%, the tensile strength and elongation at break can reach 8.38 MPa and 26.72%, which is better than traditional cellulose membranes. Finally, the separation properties were studied by separation BSA from water. The results showed that the rejection rate and water flux changed positively and negatively with the change of MCC content. When the content was 5%, the membrane demonstrated the best comprehensive performance, its rejection for BSA was 37.23 g/(m^2^ h), the corresponding rejection rate and water flux were 88.87% and 41.89 L/(m^2^ h) respectively.

## Introduction

*Rabdosia rubescens*, a perennial herb of *Labiatae* and *Plectranthus*, is an important traditional Chinese medicine. *R. rubescens* grows widely in Taihang Mountains, Funiu Mountains and Dabie Mountains. Among them, Jiyuan county in Henan Province has the best quality of *R. rubescens*, which is bred artificially in a large quantities^1^. In the past, *R. rubescens* were always consumed as a tea for its health benefits^2^. In 1972, the esophageal cancer research center found that *R. rubescens* had a unique anticancer effect, which then has been widely used in clinical practice^3^. *R. rubescens* has the functions of clearing away heat and detoxification, anti-bacterial, promoting blood circulation, relieving pain, preventing and fighting cancer. Thus, it is well known as Penicillin in traditional Chinese medicine^4,5^. In recent years, there have been many studies on the application of the single drug and its chemical constituents in *R. rubescens.*

The medicinal part of *R. rubescens* are stem and leaf. In pharmaceutical factories, they mainly use ethanol aqueous solutions to extract pharmaceutical ingredients^6^. Statistically speaking, one kilogram of hay can obtain 0.1 to 0.12 kg extract, above 80% of the total extraction residue becomes solid waste, causing a waste of resources. The results from our previous study showed stem residue of *R. rubescens* is rich in cellulose, and its fiber quality is also excellent. If the stem residue is properly used, it is highly possible to turn waste into valuable resource. So, how to use the residue valuably is a meaningful research project.

It is well known that cellulose membranes have the characteristics of wide sources, low cost, biodegradability, diverse properties and easy chemical modification, which have become one of the important development directions of functional membrane materials in recent years. In the process of preparing membrane materials, the quality of cellulose raw materials has important effect on the performance of membrane materials.

Among the numerous celluloses raw materials, microcrystalline cellulose (MCC) is a naturally occurring substance obtained from purified and partially depolymerized cellulose. It is a white particle with an average grain size of 20–80 μm, low polymerization degree^7^, high crystallinity^8^, and large specific surface area^9^, so it is more suitable for preparing regenerated cellulose membranes. At present, the most commonly used method for preparing MCC is acid hydrolysis. In response to the problems of high acid concentration causing equipment corrosion, difficult treatment of waste liquid, and large water consumption. Although researchers have attempted to develop some new acid hydrolysis methods, such as ultra-low acid hydrolysis methods, there always had more or less disadvantages of expensive but low active catalyst, higher temperature and difficulty of product seperating^10,11^. Meanwhile, MCC also suffers some drawbacks in properties that are desired for some applications, such as, poor wettability, moisture absorption, incompatibility with most polymeric matrices and limitation in processing temperature^12^. Therefore, the selection of appropriate raw materials, good preparation process and effective additives are very important to the performance of the membrane. Although there are currently reports that microcrystalline cellulose prepared with corn cob^13^, banana tree waste^14^, tea residue^15^, jute^16^ and oil palm fiber^17^ as fillers used in the preparation of cellulose membranes. However, jute and oil palm fiber are expensive, and the supply of tea residue and banana tree waste is difficult. Corncobs are mainly used as feed, and their promotion is subject to certain restrictions. The *R. rubescens* residue does not have the above problems and can be used as a rich and high-quality source of cellulose. At the same time, the research on the preparation and performance of *R. rubescens* MCC and its membrane materials has not been reported before. In this paper, the conventional cellulose was treated by acid-catalyzed hydrolysis to obtain MCC. Depending on the intrinsic properties of MCC to overcome the shortcomings of conventional cellulose membrane such as low strength and uneven membrane pores, a cellulose membrane with better comprehensive properties was successfully prepared.

## Materials and methods

### Extraction of *R. rubescens* cellulose

*Rabdosia rubescens* cellulose was extracted and purified according to the literature^18^.

### Preparation of *R. rubescens* MCC

2.00 g *R. rubescens* cellulose was mixed with 40 mL hydrochloric acid of certain concentration thoroughly in a 250 mL three mouth flask with mechanical agitatora and condenser, and then was carried out at a certain temperature for a period of time. When the reaction was terminated and cooled to room temperature, the mixture was filtered in vacuum and washed with distilled water to neutrality. Then dried at 60 °C to constant weight in a vacuum drying oven and crushed to obtain white microcrystalline cellulose powder.

In order to obtain the optimized operational conditions, according to the references^19^, the orthogonal experiment was designed and carried out on the basis of pre-experiment. The selected experimental factors were Hydrogen chloride concentration (A), reaction temperature (B) and hydrolysis time (C) respectively. And the levels of factor A, B and C were designed and shown in Table 1.

**Table 1:** The designed levels of the reaction factors.

Level	A (%)	B (°C)	C (min)
1	5	60	60
2	10	70	70
3	15	80	80

### Preparation of *R. rubescens* MCC membrane

Under vacuum condition, the marketed 50% *N*-methylmorpholine-*N*-oxide (NMMO) aqueous solution was concentrated at 55 °C until 86.5% concentration. Then 0.5% propyl gallate was added to the solution, shaken to store in a cool and dark place for later use^20^.

The MCC was added to the NMMO concentrated aqueous solution in a certain proportion, swelled in the reactor for 3–4 h at 100 °C, and then mechanically stirred until the cellulose was completely dissolved, forming a translucent amber gelatinous liquid. Let stand for 4 h at 100 °C for deaeration, pour the solution on a thermostatic glass plate at 85 °C, and scrape the membrane at a constant speed with an I-shaped coater (Specification: 500 μm). After scraping the membrane, quickly put it in the deionized water as coagulation bath for 24 h and change water every 8 h. The solidified *R. rubescens* officinalis MCC membrane was put into a glycerin aqueous solution (C_3_H_8_O_3_:H_2_O = 30 mL:100 mL) for plasticization for 1 h, then taken out, the surface moisture was absorbed by filter paper, and then placed in an vacuum drying oven at 60 °C for drying to constant weight.

### FTIR analysis

The samples were vacuum-dried at 80 °C for 12 h, then were ground and compressed with KBr powder, and scanned and analyzed from 4000 to 400 cm^−1^.

### XRD analysis

X-ray diffraction equipped (D8, Bruker, Germany) with Cu Kα radiation in the 2θ range 3–50° with step size of 0.02°, was used under the operational conditions of 40 kV and 40 mA, scanning speed 10°/min. Crystallinity index can be calculated from the height ratio between intensity of crystalline peak and total intensity of non-crystalline peak using the formula below^21^.

1 $$ CrI = \frac{{(I_{002} - I_{{{\text{am}}}} )}}{{I_{002} }} \times 100\% $$

where, the *CrI* is crystallinity index, *I*_002_ is maximum intensity of the peak, and *I*_am_ is intensity of diffraction of the non-crystalline material.

### Thermal analysis

6 mg sample was put into an alumina crucible, which was heated from 25 °C to 500 °C under the condition of nitrogen flow rate of 20 mL/min, and the heating rate was 10 °C/min.

### Mechanical performance test

The membrane samples were cut into a rectangle of 2.0 × 6.0 cm, and the mechanical properties were tested on a universal tensile testing machine. The tensile speed was 50 mm/min and the clamp was 50 mm. The testing method was referred to GB/T1040.3-2006 of China.

### Separation performance test

The membrane was cut into a circle with diameter of 4 cm, and measured with a prepared 1.00 g/L bovine serum albumin (BSA) aqueous solution, pre-pressured at 0.2 MPa for 20 min, and then set the pressure to 0.1 MPa for 10 min, and the measurement was collected. The membrane’s permeating flux was calculated by formula (2)^22^, the interception rate was calculated by formula (3), and the interception flux was calculated by formula (4), the BSA concentration of the permeate was measured by a spectrophotometer, and compare it with the original solution to obtain the rejection rate^23^.

2 $$ J = \frac{V}{St} $$

*J*: water flux, L/(m^2^ h); *S*: the effective area of the membrane, m^2^; *V*: the volume of liquid passing through the membrane in a certain time, L; *t*: the test time, h.

3 $$ R = \left( {1 - \frac{{C_{1} }}{{C_{0} }}} \right) \times 100\% $$

*R*: rejection rate, %; *C*_0_ : concentration of BSA in the stock solution, g/L; *C*_1_: concentration of BSA in the permeation solution, g/L.

4 $$ Q = JC_{0} R $$

*Q*: interception, g/(m^2^ h).

## Results and discussion

### Optimization of MCC preparation process by response surface methodology

According to the model designed by response surface as shown in Table 2, the main breaking zone of hydrolysis reaction is in the amorphous region of cellulose, the non-crystalline part was removed, which increases the crystallinity of cellulose and obtains more purified MCC. Our pre-studies have shown that the hydrolysis of cellulose requires much energy because of its large activation energy^15,24,25^. But if the energy is too high, the glycosidic bond of cellulose will be broken and hydrolyzed to soluble glucose, which will cause MCC loss directly. Meanwhile, if reaction time is excessively extended, the crystalline area of cellulose will also suffer a certain degree of damage. So, there have appropriate values for hydrogen chloride concentration, reaction time and temperature.

**Table 2:** Results and analysis of designed orthogonal experiments.

No	A	B	C	Yield (%)
1	1	1	1	92.08
2	1	2	2	94.95
3	1	3	3	91.25
4	2	1	2	95.48
5	2	2	3	92.34
6	2	3	1	92.95
7	3	1	3	91.07
8	3	2	1	91.03
9	3	3	2	88.29
K_1_	278.28	278.63	276.06	
K_2_	280.77	278.32	278.72	
K_3_	270.39	272.49	274.66	
R	10.38	6.14	4.06	

According to the results in Table 2, the influence order of the three factors is R(A) > R(B) > R(C). It shows that the concentration of HCl has the largest effect on hydrolysis reaction and should be gained especially attention. In fact, Hydrogen chloride acts on catalyst as a result of its strong acidity, not just the solvent. The best factors combination is A2B1C2, the corresponding yield of MCC is 95.48%. According to this result, three parallel experiments are carried, and the average yield of MCC is about 95.03% that is closed to the theoretical yield.

### FTIR analysis

**Figure 1:**
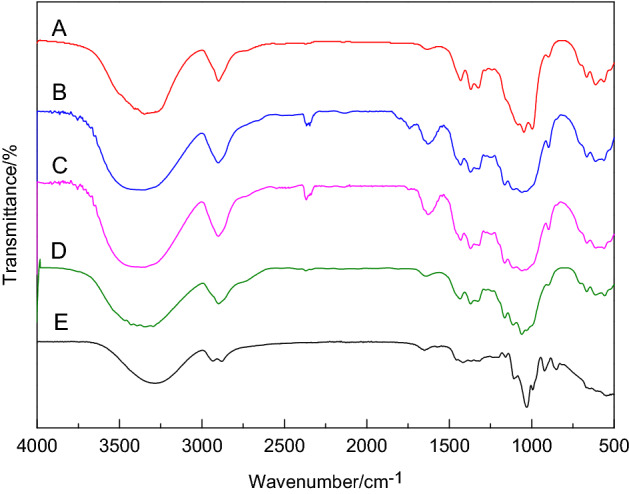
FTIR spectrum of A (cellulose standard sample), B (Rabdosia cellulose), C (*R. rubescens* MCC), D (MCC standard sample) and E (MCC membrane).

As shown in Fig. 1, it can be seen from the FTIR spectrum that they all have the absorption peaks at 3400–3360 cm^−1^, 2930–2900 cm^−1^, 1634–1590 cm^−1^, 1370–1330 cm^−1^, 1050–1030 cm^−1^, and 900–890 cm^−1^ in common represent the –OH stretching vibration peak, the –CH stretching vibration peak, and –C=O stretching vibration peak, –CH bending vibration peak, –C–O–C stretching vibration characteristic peak and alienation β-bond^26^. It can be seen that the position of the basic characteristic peak of cellulose has not changed, which indicates that the molecular structure of the MCC was basically unchanged during the process of hydrolysis and the quality is basically consistent with the commercial MCC standard sample. It can be seen from the figure that the –OH stretching vibration peak of the cellulose membrane at 3445 cm^−1^ shifts to 3389 cm^−1^, indicating that the hydroxyl groups are rearranged after the cellulose membrane is dissolved and regenerated, and the hydrogen bond binding force decreases to produce a red shift. The absorption peak at 1020 cm^-1^ becomes sharp, indicating that the C–O in the amorphous region becomes stronger after regeneration and the cellulose crystal form has changed from type I to type II. The absorption peak at 898 cm^−1^ is that the amorphous band of the cellulose film becomes stronger and the degree of amorphous becomes larger^27^.

### XRD analysis

**Figure 2:**
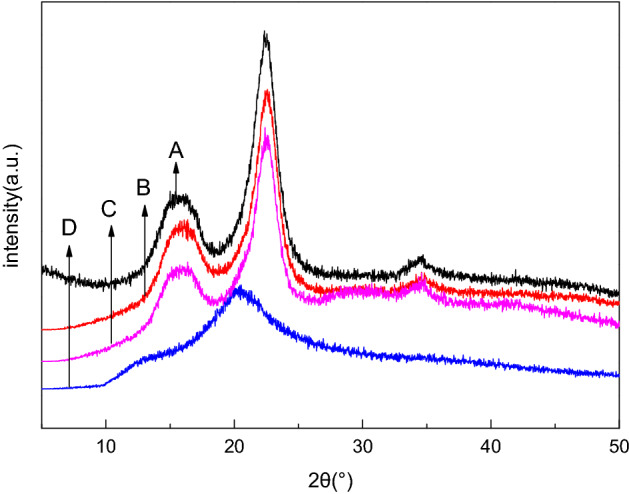
XRD spectrum of A (standard MCC sample), B (*R. rubescens* MCC), C (Rabdosia cellulose) and D (MCC membrane).

It can be seen from Fig. 2 that the positions of the diffraction peaks of Rabdosia cellulose, the *R. rubescens* MCC and standard MCC sample are the same, and diffraction peaks appear at 15.4°, 22.5°, and 34.6°, and their crystal structure is type I according to the interrelated theory^28^, indicating the preparation of MCC is basically as same as the MCC standard sample. Calculated with Segal formula, it is found that the crystallization index of MCC is slightly lower than MCC standard sample. The MCC membrane has a diffraction peak at 20.8°, and its crystal structure is type II^29^, indicating that the crystalline structure of cellulose has changed during the course of dissolution and precipitation in NMMO/H_2_O. After the membrane was formed, the diffraction intensity decreases, and the crystallinity index drops to 39.37%, which is attributed to the strong polar oxygen atoms on the N–O in the NMMO attacking the hydrogen bond and breaking it during the dissolution process.

### Thermal analysis

**Figure 3:**
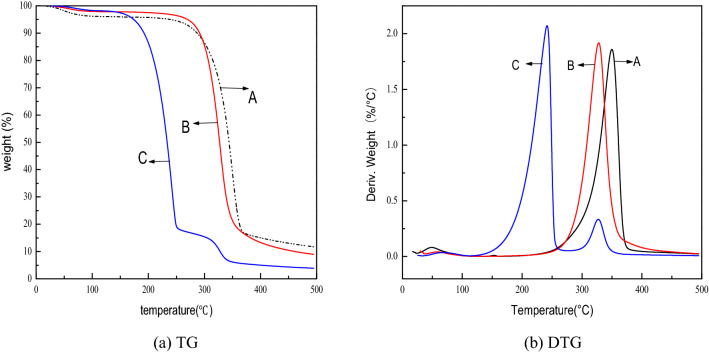
TG and DTG curve of A (standard MCC sample), B (*R. rubescens* MCC) and C (MCC membrane). (**a**) TG, (**b**) DTG.

The thermal curve of standard MCC sample, Rubescens rubescens MCC and *R. rubescens* MCC membrane measured at a heating rate of 10 °C/min are shown in Fig. 3. The thermal decomposition behavior of the samples can be roughly divided into three intervals. The first stage is the slight weight loss stage, which is mainly manifested by the volatilization of intermolecular bound water and additives. The second stage is the thermal decomposition stage which can cause a significant weight loss. The third stage is the stability of carbon formation, the sample has been basically carbonized at this stage, and the increase in temperature has relatively small effect on the weight loss of the residue^30^.

From Fig. 3a, compared with the standard sample, the thermal weight loss of *R. rubescens* MCC has similar curve. In the first stage, the weight loss rate of *R. rubescens* MCC is slightly lower than the standard sample, indicating that it is more hydrophilic than the standard sample. The initial pyrolysis temperature is about 275 °C, which is basically as same as the standard sample. It can be seen from the Fig. 3b that its maximum weight loss rate temperature is 327 °C, which is 22 °C lower than the standard sample, and the remaining residue rate was 8.9%. Overall, the thermal stability of *R. rubescens* MCC is not much different from that of standard MCC sample. In sharp contrast, the initial pyrolysis temperature of *R. rubescens* MCC membrane is about 151 °C, and the maximum pyrolysis rate temperature is 218 °C. The thermal stability after membrane formation is lower than before membrane formation, which may be attributed to the dissolution and regeneration process. The hydrogen bond between the cellulose is not completely rebuilt after it was opened in NMMO/H_2_O, and the decrease in crystallinity after dissolution also affects the thermal stability of the MCC membrane^22^.

### Influence of MCC content in casting solution on the mechanical properties

**Figure 4:**
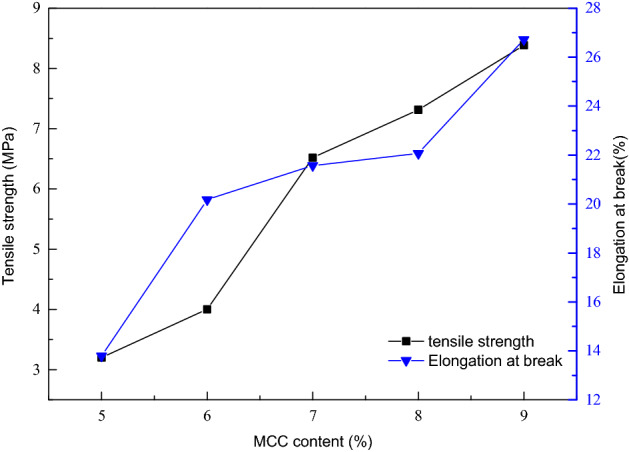
Effect of MCC content in casting solution on mechanical properties of the membrane.

Figure 4 presents the influence of the content of MCC in casting solution on the mechanical properties of the MCC membrane. It can be seen that with the increase of the content, the tensile strength and elongation at break of the MCC membrane both show an increasing trend. When the content of MCC increases from 5 to 9%, the tensile strength of the membrane increases from 3.20 MPa to 8.38 MPa and the elongation at break increases from 13.79% to 26.72% respectively. It may be due to the increase in cellulose content in the casting solution ultimately. Because of the increase as the molecule numbers in per unit volume, extrusion with each other can be occurred, which can increase the intermolecular microcrystalline entanglement. At last, the tighter intermolecular connection improves mechanical properties of the membrane. However, it should be noted that as a functional separation membrane, the content of casting solution should not be too high, otherwise the membrane body will be too dense, which will reduce the permeability significantly and even make it losing the separation function.

### Influence of MCC content in casting solution on the membrane’s hydrophilicity

The contact angle is one of the indexes to measure the hydrophilic or hydrophobic properties of the membrane. The hydrophilicity or hydrophobicity of the membrane has a certain influence on application field of the membrane.

**Figure 5:**
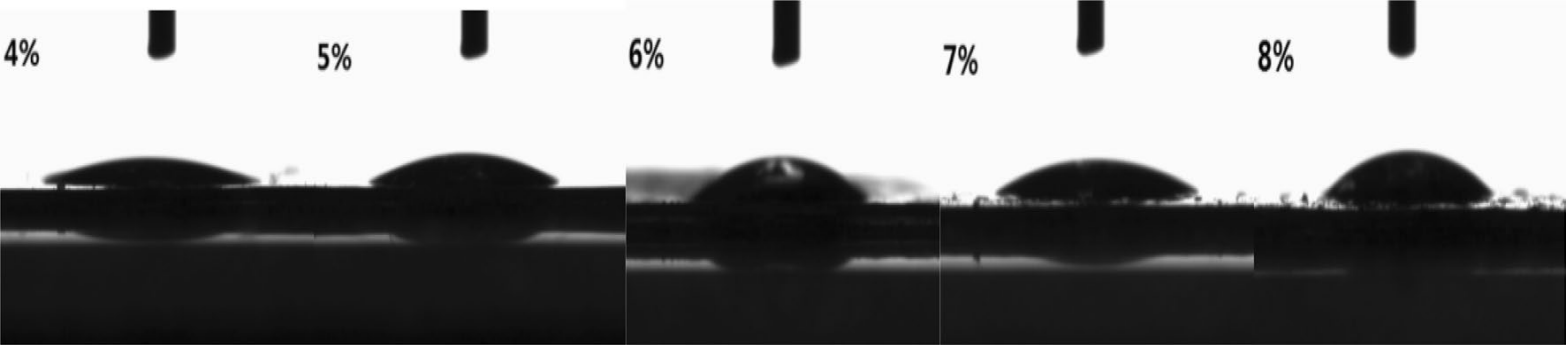
Contact angle photo of membranes prepared from casting solutions with different MCC content.

Therefore, it is of great significance to control the hydrophilic and hydrophobic properties of the membrane. From perspective of cellulose structure containing hydrophobic carbon ring and hydrophilic hydroxyl group, so cellulose molecule should have certain amphiphilic property. Therefore, hydrophilic and hydrophobic properties of the membrane should be controllable in some ways. Our research team has determined the membranes formed by different cellulose contents in casting solution, finding that the contact angles of water against membrane are also different. The contact angles are shown in Fig. 5 and Table 3. The data in Table 3 show that when the content of cellulose in casting solution increases, the contact angle increases concomitantly, indicating that the hydrophobicity of the cellulose membrane increases simultaneously. This is because the higher the content of microcrystalline fibers in the casting solution, the denser the membrane is. Meanwhile, the shrinkage of the membrane pores causes a smaller specific surface area and less exposed hydroxyl groups, which can reduce the hydrophilicity of membrane to a certain extent.

**Table 3:** Contact angle of membranes with different MCC content in casting solution.

MCC content (%)	4	5	6	7	8
Contact angle (°)	18.6	24.7	29.4	31.0	36.8

### Effect of MCC content in casting solution on the separation performance of membrane

**Figure 6:**
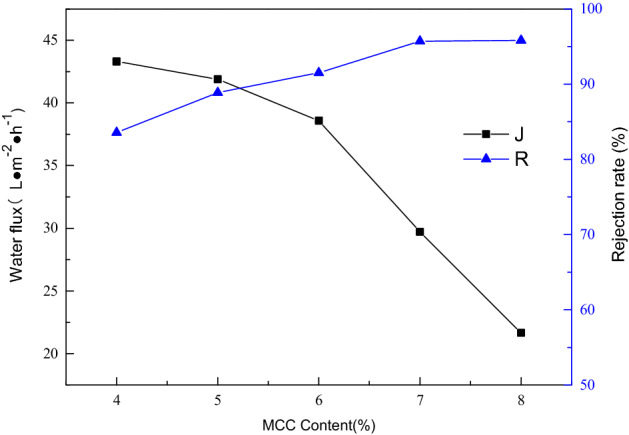
Effect of MCC content in casting solution on the water flux (J) and rejection rate (R) of the membrane.

The water flux and rejection rate of membrane are both important indicators to characterize the performance of the membrane. Here, the flux and rejection rate of the membrane formed by the casting liquid with different MCC content to 1.0 g/L bovine serum albumin (BSA) aqueous solution were determined under the pressure of 0.1 MPa. The results are presented in Fig. 6

.

It can be seen from Fig. 6 that under the same pressure, with the increasing of the cellulose content in the casting solution, the water flux of the formed membrane shows a downward trend, but the rejection rate continues to increase. When it reaches 7%, the water flux still declines but the rejection rate hardly changes. This phenomenon is due to the increase in the force among cellulose molecules as a result of increase in cellulose content, which will be more tightly bonded to each other when forming a membrane. So, the membrane pore size will become smaller and reduce the water flux. Those results are almost consistent with the previous conclusion that the higher the cellulose content in the casting solution, the stronger the hydrophobicity of the cellulose membrane is, which is accompanied by a certain exclusion effect on water.

Therefore, selecting the appropriate cellulose content is of great significance to the separation effect of the regenerated cellulose membrane. Here, under a certain pressure, the solute rejection amount per unit time and unit area (Q) of the membrane represents the separation efficiency of the cellulose membrane.

**Figure 7:**
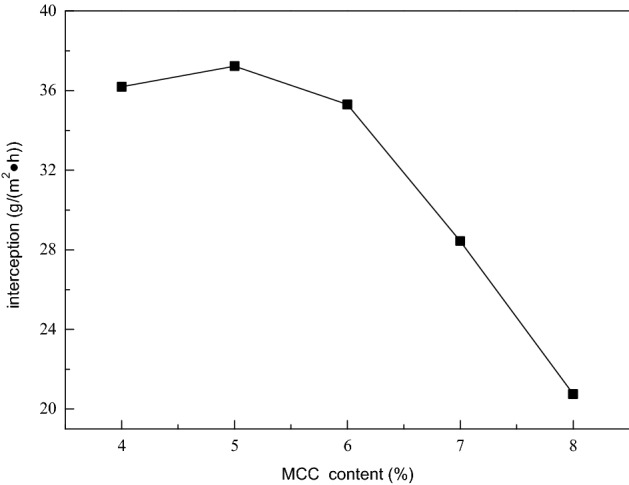
Effect of MCC content in casting solution on the Q value of the membrane.

It can be seen from Fig. 7 that the cellulose membrane formed by casting solution with 5% cellulose content has the highest rejection amount of BSA, and its value can reach 37.23 g/(m^2^ h).

### The digital picture of the film

**Figure 8:**
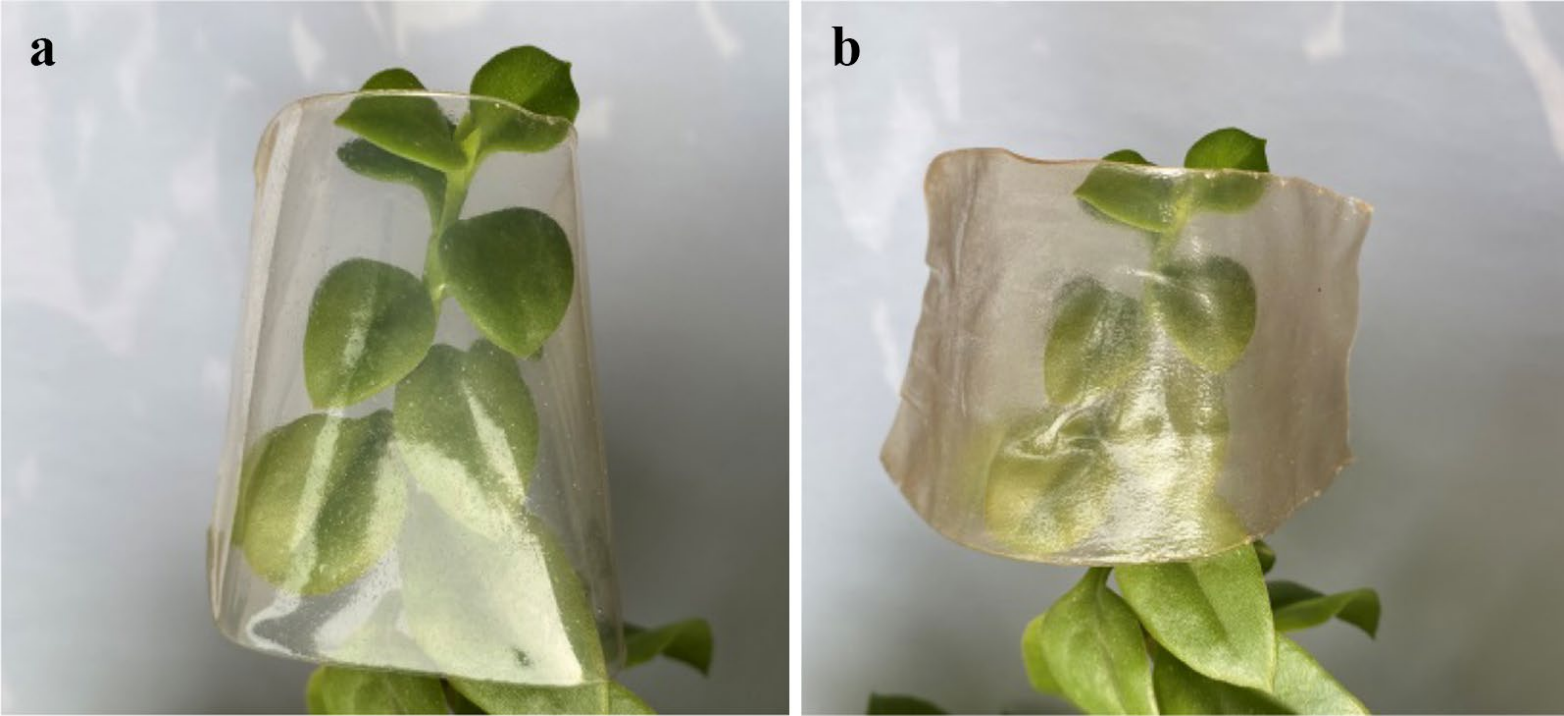
Digital photos of film with 5% (**a**) and 8% (**b**) MCC content in casting solution.

The cellulose membranes prepared by casting solution with 5% and MCC content were photographed by a digital camera. The photos are demonstrated in Fig. 8.

Figure 8 shows that the film with 5% MCC content is flat, smooth and transparent, while the other is wrinkled and the transparency decreases. Theoretically, as the cellulose concentration increases, the viscosity of the casting solution gradually increases. When the cellulose concentration is too low, the number of cellulose molecules per unit volume is small, the interaction force is weak, and the fluidity is too large, so it is difficult to form a film. On the other hand, if the cellulose concentration is too large, the viscosity of the casting film is too high and the fluidity becomes poor, resulting in uneven film formation. 5% content is appropriate for casting solution in practice.

### SEM analysis of the membrane

**Figure 9:**
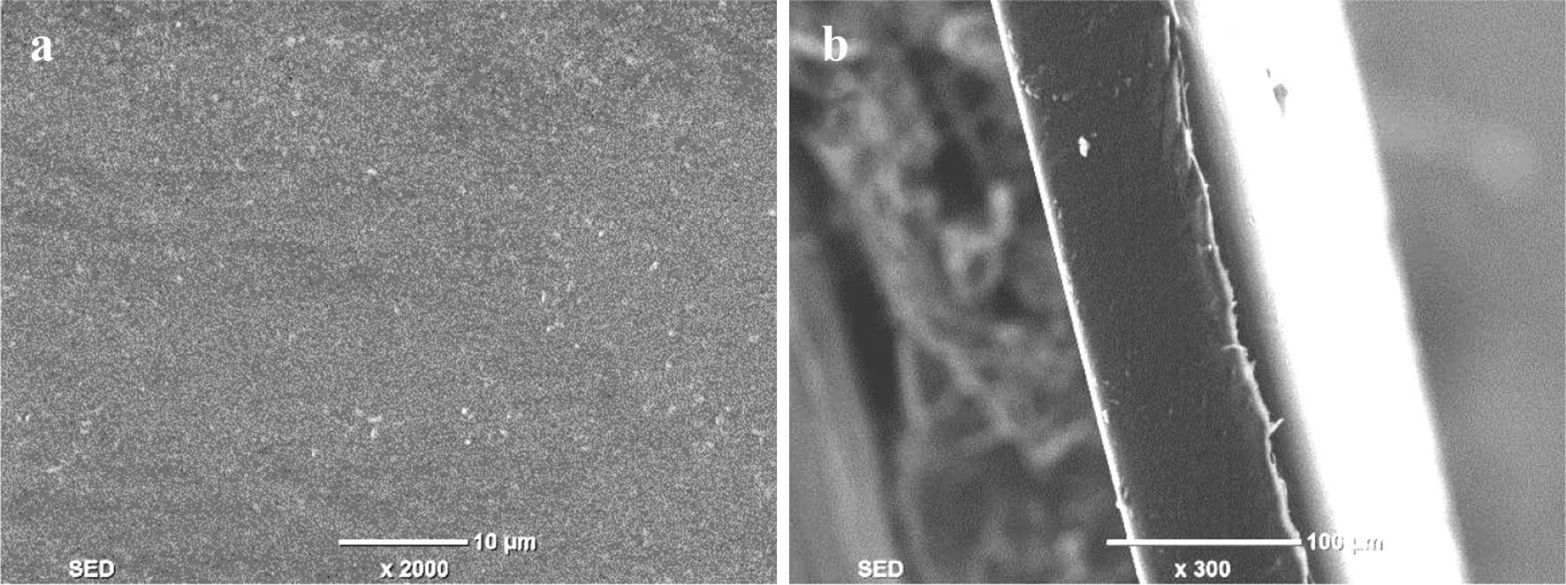
SEM images (floor plan (**a**) and cross section (**b**)) of the membrane prepared by casting solution with 5% MCC content.

The cellulose membrane prepared by casting solution with 5% MCC content has the relative best separation performance, so it was taken as the test object, and its microstructure was analyzed using SEM analysis. The plane and section structure are shown in Fig. 9.

It can be seen from Fig. 9a that the surface of membrane is smooth, flat and uniform without obvious structural defects in general. As can be seen from Fig. 9b section, the section of the membrane shows a high density spongy shape, indicating the bondings among the cellulose molecules are combined tightly. So, the membrane can show a high mechanical strength and separation performance.

## Conclusions

In this paper, MCC of *R. rubescens* residue was prepared via hydrolysis in dilute hydrochloric acid and the yield of MCC was about 95.03% under the optimized conditions. Then, the MCC membrane was prepared from MCC and characterized by FT-IR, SEM, XRD, TG and DSC. The results show that the crystalline form of cellulose changes from cellulose I to cellulose II in the preparation of cellulose membrane. At the same time, the thermal stability of cellulose decreases greatly. It was also found that the content of MCC in the casting solution has a great influence on the mechanical properties of the MCC membrane, and the tensile strength and elongation at break increase with the concentrating of MCC in casting solution. It is worth noting that the relationship between membrane separation performance and MCC content is more complicated. The rejection rate and water flux change with MCC content positively and negatively. When water flux increases from 21.66 L/(m^2^ h) to 43.31 L/(m^2^ h), the rejection rate drops from 95.80% to 83.56% correspondingly. So, in practical application, it is necessary to choose a suitable balance between rejection rate and water flux to meet the requirements of the specific process aiming to a satisfied result.
